# Going stressed underground: corticosterone-induced stress responses alter the movement and spatial ecology of males in a fossorial reptile

**DOI:** 10.1186/s40462-026-00655-9

**Published:** 2026-05-07

**Authors:** José Martín, José Javier Cuervo, Pablo Recio, Gonzalo Rodríguez-Ruiz, Isabel Barja, Pilar López

**Affiliations:** 1 Departamento de Ecología Evolutiva, Museo Nacional de Ciencias Naturales, CSIC, José Gutiérrez Abascal 2, Madrid, E-28006 Spain; 2 College of Science and Engineering, Flinders University, Bedford Park, Australia; 3 Climate Research Foundation - Fundación para la Investigación del Clima, Madrid, Spain; 4 Laboratorio de Eto-Ecofisiología, Departamento de Biología (Unidad Zoología), Facultad de Ciencias, Universidad Autónoma de Madrid, Madrid, Spain; 5 Centro de Investigación en Biodiversidad y Cambio Global (CIBC-UAM), Universidad Autónoma de Madrid, Madrid, Spain

**Keywords:** Amphisbaenians, Glucocorticoids, Fossoriality, Stress response, PIT-tag telemetry

## Abstract

**Background:**

Increased levels of physiological stress caused by environmental perturbations may alter an individual’s physiological condition, which in turn may influence its movement and space-use strategies. These effects can vary depending on the environment, context, and lifestyle of each species. However, some animal groups, such as fossorial species, have been rarely examined, even though numerous environmental and anthropogenic factors can strongly affect the quality of the underground soil environment they inhabit.

**Methods:**

We examined how an aspect of the stress response affected the movement and spatial ecology of males in a strictly fossorial reptile, the amphisbaenian *Blanus cinereus*. Males were subjected to repeated, non-invasive corticosterone (CORT) supplementation, the main hormone mediating the stress response in reptiles. Then, we used passive integrated transponder (PIT) telemetry to track for 14 days the movements and space use of CORT-supplemented and control males, as well as non-CORT manipulated females, while moving undisturbed underground in outdoor seminatural enclosures. We hypothesized that experimentally elevated CORT would alter movement behavior, particularly reducing activity and space use in males.

**Results:**

The CORT treatment decreased underground movement activity, distances moved, number of movements, and daily areas covered by CORT-supplemented males. However, although all males were in close proximity to females more often than toeach other, there was no effect of CORT supplementation of males on their spatial relationships (distance and proximity) with male and female conspecifics.

**Conclusions:**

Repeated elevation of CORT may modify the underground movements and space use of fossorial reptiles, which may represent a strategy to compensate for the effects of glucocorticoid activation on physiological condition and energetic allocation, avoiding predation risk or the energetic costs of burrowing. Our study contributes to understanding the effects of soil environmental perturbations, such as pollution or drought, on the ecology of fossorial animals, and how these species might cope with these potential stressors.

## Background

When animals move for foraging, searching for mates, or dispersing, they do not move through and use all the available space randomly, but follow ecological patterns that are characteristic of each species [1, 2]. Movement and space-use strategies also vary due to ecological constraints, such as environmental factors (e.g., climate or the distribution of resources) or the density of conspecifics or predators [3, 4]. Variations may also occur due to sex, age, or physiological constraints, such as energetic, foraging, and reproductive requirements, or the ability to move in certain habitats [e.g. [5–8].

Environmental disturbances can alter an individual’s physiological condition, particularly by affecting energy allocation, immune investment, and metabolic balance. Such changes might also influence its movement and space use strategies. For example, situations such as adverse weather, food deprivation, or human disturbances often induce the secretion of glucocorticoid hormones (GCs), such as corticosterone (CORT) in many vertebrates [9, 10]. This increase in CORT ultimately leads, in the short-term, to several positive physiological and behavioral adjustments aimed at avoiding or balancing the stressors’ detrimental effects [11, 12]. However, if the perturbation is repeated or persists for a long time, the sustained physiological stress response may result in an allostatic load, leading to potential physiological costs, such as altered energy allocation or reduced immune investment [13, 14]. Nevertheless, the effects of increased CORT may vary among individuals [15] and be dose- and ecological context-dependent [10, 16–18], rather than reflecting an inherently detrimental state.

In addition to mediating stress responses, circulating CORT also reflects individual variation in metabolic rate, independent of acute stressors [19]. This link between GCs and baseline energetic demands provides a mechanistic basis for expecting CORT-induced shifts in movement and space-use behavior. However, studies linking the physiological stress response to aspects related to the spatial and movement ecology of animals, such as activity, home range, and dispersal, sometimes show inconsistent results. For example, CORT supplementation increased perch hopping in white-crowned sparrows [20] and raised locomotor activity in two species of lizards [12, 21]. In contrast, CORT supplementation reduced activity, home range size, and aggressiveness in male side-blotched lizards [22–24]. Also, repeated human handling decreased the locomotor activity of a terrestrial salamander, although this was unaffected by acute increases in supplementary CORT [25]. Furthermore, CORT did not affect the activity of white-crowned sparrows exposed to short photoperiods [16] and did not influence dispersal of adult willow tits either [26]. Consequently, elucidating the effects of increased stress responses on animal movement and spatial ecology necessitates a broad comparative framework encompassing varied stressors and species with diverse ecological adaptations and lifestyles. In practice, however, certain groups, such as fossorial animals, are often understudied due to the difficulties associated with locating and working with them [27, 28], even though numerous environmental and anthropogenic perturbations may alter the quality of the underground soil environment they inhabit [29]. Soil alterations may lead to potentially stressful situations that can negatively affect fossorial animals [30–32] but often remain unnoticed, resulting in a threat to biodiversity.

Among reptiles, amphisbaenians are highly adapted morphologically, physiologically, and ecologically to an underground life [33, 34]. The amphisbaenian *Trogonophis wiegmanni* shows high site fidelity, moving short distances and over small areas, and spending some days without any noticeable movement, even under favorable environmental conditions [35]. This movement and spatial strategy may be driven by the energetic costs of burrowing [7], or to the low metabolic demands of fossorial reptiles [36]. As a result, they cover much shorter distances and smaller areas than similarly sized epigeal species [5]. This limited mobility of amphisbaenians may make them more vulnerable to local soil alterations. For example, soil pollution by heavy metals and prolonged droughts can increase GC levels in fossorial amphisbaenians [32, 37], but the effects of elevated GC levels on their underground spatial and movement strategies remain unknown.

Here, we examined how an aspect of the stress response (i.e., elevated CORT) affect the movement and spatial ecology of a strictly fossorial reptile, the amphisbaenian *Blanus cinereus*. More specifically, males were subjected to repeated non-invasive CORT supplementation [38]. This is the primary GC in reptiles and plays a central role in regulating energy allocation, locomotor activity, and behavioral adjustments to environmental challenges [9]. Importantly, repeated activation of GC pathways can generate behavioral carry-over effects that persist beyond the period of hormone elevation, even without evidence of a chronic physiological stress response. Understanding these short-term but lasting behavioral consequences is essential for predicting how fossorial species may adjust their movement strategies following environmental disturbances that elevate CORT.

After the supplementation, we placed together several CORT-supplemented and control males, as well as not CORT manipulated females in outdoor seminatural enclosures and followed the underground movements of undisturbed individuals using passive integrated transponder (PIT) telemetry (i.e., detecting at a distance the radiofrequency signal of PIT-tagged buried individuals) [35, 39–41]. Over 14 days, we obtained data on their short-term movement patterns and locations in the enclosure, as well as their spatial relationships with other individuals during the day. Because prolonged elevations of CORT can shift metabolic and energetic allocation away from processes such as immune function or reproduction, individuals may adjust their behaviour to prioritise immediate survival. Therefore, we predicted that males experiencing experimentally elevated CORT will adjust their movement and space-use strategies, either by reducing activity or by modifying the extent of space explored in order to conserve energy. CORT-supplemented males would also avoid being close to other males to elude costly agonistic intrasexual interactions. Additionally, the restricted movements of CORT males could reduce the rate of encounters with females, and, moreover, females might show spatial avoidance of CORT-supplemented males, which could occur if elevated CORT altered male chemical cues associated with quality or physiological condition [42].

## Methods

### Study animals and maintenance

The amphisbaenian *Blanus cinereus* is a strictly fossorial reptile endemic to the Iberian Peninsula [43]. It lives buried in the soil in areas with sandy substrates and abundant leaf litter [44]. Fieldwork was conducted in April 2021, which corresponds to the onset of the reproductive season for this species [43]. This timing was chosen deliberately, as individuals are more active and easier to capture during this period, and movement patterns are ecologically meaningful due to increased reproductive activity. We captured 30 adults *B. cinereus* by lifting stones in an oak forest near Navacerrada (40º43’N, 04º01’W; Madrid, Spain).

After capture, amphisbaenians were transported to the “El Ventorrillo” MNCN-CSIC field station (5 km from the capture site), where they were measured (20 males, snout-to-vent length, SVL, mean ± SE = 194 ± 3 mm; 10 females, SVL = 202 ± 5 mm; one-way ANOVA on log-transformed SVL males vs. females, F_1,28_ = 2.51, *p* = 0.12). Before starting the experiment (between 15 and 20 days after capture), we kept the amphisbaenians at the field station individually in indoor plastic terraria (40 × 30 × 30 cm) with a 5 cm layer of coconut fiber substrate, where amphisbaenians remained buried all the time, and with a 15 × 15 cm flat tile over the surface simulating a rock, under which they could thermoregulate and forage. Mealworm pupae (*Tenebrio molitor*) dusted with vitamin powder were provided as food beneath the tiles three times a week. Water was provided daily by spraying to maintain drinking water availability and substrate moisture close to the preferred levels of this species (i.e., mean relative soil moisture = 13%) [37]. A heating cable running under the terraria, connected to a thermostat programmed to 22 °C during daylight hours, maintained soil temperature close to the preferred level of these amphisbaenians (i.e., mean substrate temperature = 20.7 ºC) [45]. Photoperiod matched natural conditions.

### PIT tag marking

Each amphisbaenian was individually marked the day after capture by subcutaneously implanting a PIT tag in the upper right side of the body (Biomark MiniHPT8; Biomark, Inc., Boise, ID, USA; length = 8.4 mm, diameter = 1.4 mm, weight = 0.03 g). This marking method does not have long-term negative effects in amphisbaenians [35, 46]. PIT-tags were useful for following undisturbed buried animals during the experiment and, subsequently, for population monitoring when animals were released at their capture sites at the end of the experiment.

### Corticosterone supplementation

We experimentally increased the circulating CORT levels of male amphisbaenians using a non-invasive method. For eight days (from 7th to 15th May), ten males were daily supplemented with 5 μL of a solution of 2 μg corticosterone dissolved in 6 mL of soybean oil, applied with a pipette to the dorsum (CORT treatment). Another ten males were supplemented with soybean oil alone (control treatment). Male amphisbaenians were randomly assigned to treatments and did not differ in body size (SVL: CORT, mean ± SE = 194 ± 4 mm; control = 193 ± 4 mm; one-way ANOVA on log-transformed SVL, *F*_1,18_ = 0.03, *p* = 0.85). The high lipid content of reptile skin allows lipophilic molecules to be quickly absorbed into the bloodstream [21]. This supplementation procedure has been shown to be effective in several species of lizards [10, 12, 21] and validated for a similar amphisbaenian species [38]. Although CORT supplementation might not perfectly mimic the full physiological stress response induced by environmental stressors [47], it provides a valuable tool to investigate a fundamental component of the stress response, the rise in circulating CORT levels and its interaction with behavior [22]. Our approach therefore aims at examining the behavioral consequences of experimentally elevated CORT, without assuming equivalence to any particular environmental perturbation.

We applied the supplementations each morning at 07:00 h GMT when amphisbaenians had low body temperatures and were inactive. Before applying the solution, we gently removed all males from their home cages and placed them individually in small empty cages inside an incubator at 10 °C for one hour. This procedure further decreased body temperature and activity levels of males, facilitating handling and reducing potential stress in control individuals. After supplementation, we maintained males under the same temperature conditions for 1 h to allow the solution to be absorbed by the skin before it could be lost by rubbing against the substrate, as active individuals quickly started to burrow once released back into their home cages.

### Outdoor seminatural enclosures

We prepared five enclosures, each consisting of an inflatable circular plastic pool 1.5 m in diameter (approx. 1.8 m^2^ of surface) and with walls 50 cm high. We added a 2 cm layer of commercial gravel at the bottom of the pool for rainwater drainage, and above it, we placed a 10 cm depth layer of a commercial sandy substrate (commonly used for construction), mixed with a commercial substrate made with organic matter (used for gardening). This mixture had similar physical characteristics to the sandy substrates with organic matter selected by *B. cinereus* in the field [44]. We used commercial substrates to homogenize the physical and chemical properties of the soils across all enclosures, avoiding potential confounding effects of small variations in natural soils collected in the wild, as well as the presence of fungi, invertebrates, and other organisms. In addition, this avoided the environmental impact that would have resulted from digging and extracting large amounts of soil from natural habitats. In each enclosure, we also placed five 15 × 15 cm flat tiles, evenly distributed on the substrate. The surface was divided with two linear grids running from one side to the other and crossing at the center of the enclosure for reference, allowing consistent location of individual positions during each spatial measurement census (see below).

We used five independent enclosures rather than a single large shared area to ensure true replication at the enclosure level, which constitutes the appropriate experimental unit for assessing treatment effects. A single enclosure would have provided only one social and environmental context, preventing estimation of among-replicate variance and limiting statistical inference. Multiple enclosures also minimized the risk of uncontrolled dominance interactions or spatial monopolization within a single large group. All enclosures were constructed to be identical in size, structure, and layout, ensuring that individuals experienced the same physical conditions across treatments. Additionally, all enclosures were placed side by side in an open sunny outdoor area of the field station, under similar conditions of insolation and temperature. When needed, we covered the enclosures with an overhead awning to avoid rain or excessive solar radiation. Because the field station was close to the capture sites, weather conditions and photoperiod were practically identical to those experienced by amphisbaenians in their original wild population. However, small differences in microhabitat conditions (e.g., soil moisture, shade, temperature) can occur naturally in outdoor setups. These factors could generate enclosure-level variation in movement or space use, which is why ‘enclosure’ was included as a random factor in all statistical models (see below).

We introduced into each enclosure two CORT-supplemented males, two control males and two females. Females without CORT supplementation were included in the enclosures to maintain natural sex ratios and social structure. Because the objective of the study was to test the effects of elevated CORT specifically in males, only males received the hormonal treatment. Keeping females untreated avoided introducing additional sources of variation while preserving a realistic social environment. Although all individuals were adults, we matched them in size to ensure that the body size of individuals within each enclosure was similar (on average, 14 mm of SVL difference between the largest and the smallest individual in a given enclosure). In addition, individuals within each enclosure had not had any previous contact in captivity and came from field locations separated by a sufficient distance (>100 m), so they could be considered unfamiliar. We placed all individuals in the enclosures on 16th May (one day after the CORT supplementation had ended) and left them for two days for acclimatization before starting to measure movements. We provided mealworm pupae as food under the tiles three times per week and water daily with a spray to maintain soil moisture.

### Movements

We made observations over 14 days with favorable weather (from 18th May to 4th June), discarding another four days within that period with cold temperatures or rain, during which reptile activity can decrease dramatically and would have confounded the results. Each day, we recorded the location of each amphisbaenian 12 times at 30 min intervals (from 08:00 h to 13:30 h GMT). This sampling period coincides with the period of maximal activity of this species [43]. To locate individuals, we detected, without disturbing the animal, the signal from its PIT tag using a Biomark HPR Plus reader (with high detection power) and subsequently used a handheld Biomark 601 portable reader for higher positional precision. To locate each animal, we carefully moved the readers above the substrate of each enclosure, covering the entire surface until the PIT tag signal was detected. Once the animal was located, we placed a survey flag on the site and used a reference grid and a metric tape to determine (to the nearest 1 cm) the spatial position of the animal in the enclosure. This procedure was repeated until all individuals in all enclosures were located.

Before every 30 min survey, we recorded substrate temperature in each enclosure with a digital thermometer. Thus, we controlled for possible differences between enclosures in potential body temperature attained by amphisbaenians, as previous studies found a strong correlation between substrate and body temperatures in this species [48]. Substrate temperatures during surveys averaged 18.2 ± 0.6 °C, similar to those preferred by *B. cinereus* in thermal gradient experiments [45]. Although temperatures varied across times of day, they did not differ among enclosures (two-way ANOVA on log-transformed data, time of day: *F*_11,44_ = 3.80, *p* < 0.0001; enclosure: *F*_11,44_ = 0.50, *p* = 0.76).

To quantify general ‘movement activity’ of each individual, we determined, for each 30 min interval, the proportion of surveys conducted over the 14 experimental days in which an individual had changed position relative to its previous location. At the end of each day, to quantify the movements of each individual between surveys, we calculated Euclidean distances (i.e. the shortest lineal distances, to the nearest 1 cm, between each pair of successive locations in ×and y coordinates: distance _1_ to _2_ = √((x_1_-x_2_)^2^+(y_1_-y_2_)^2^)). These distances might not always represent the actual total distance moved by an individual in a given time period, as individuals might make multiple movements and follow non-linear paths between surveys. However, we considered this a very good approximate index of movement, as previous studies in terraria showed that amphisbaenians exhibit relatively low movement activity, remaining motionless for long periods at the same location (pers. observ. and see Results). For each day, we then calculated the ‘total distance moved’ during that day by each individual, by summing all distances between the 12 daily surveys. We also calculated the total ‘number of movements’ as the number of changes in location from one survey to the next in each day (i.e., if an individual was relocated at the same position, it was considered not to have moved since the previous survey). We further calculated the mean ‘distance moved per movement’ (i.e., ‘total distance moved’/‘number of movements’). For subsequent statistical analyses, at the end of the experiment, we calculated for each individual its average daily values of ‘total distance moved’, ‘number of movements’, and ‘distance moved per movement’, considering the daily values from all 14 days of the experiment.

### Space use

Every day, we assessed the ‘daily area covered’ by individual amphisbaenians while they moved undisturbed underground. We used all the spatial locations of an individual in a day (*n* = 12) to calculate the surface area of a minimum convex polygon (MCP) [49, 50]. However, we did not consider this area to be equivalent to a natural home range, given the limitations of this estimation method and because it was not measured in the field [51]. Nevertheless, even considering the apparently limited surface available in an enclosure, preliminary data showed that amphisbaenians moved within a day through areas much smaller (see also Results) than the maximum potential area (i.e., if they had used all the available space). For analyses, we calculated an average ‘daily area covered’ for each individual considering its values across the 14 days of the experiment. Finally, we also estimated the ‘total area covered’ during the 14 days of the entire experiment using all the locations of each individual (*n* = 168 locations per individual) to calculate the surface area of a MCP.

### Spatial relationships between individuals

We used the location data of individuals at each survey to calculate, as above, the linear ‘separation’ distance between each of the possible pairs of individuals (i.e., *n* = 15 pairs in each enclosure) at that survey and calculated daily average distances for each pair of individuals. In addition, as a proxy for social associations [52, 53], we estimated a spatial ‘proximity’ index between individuals. This was calculated as the percentage of times in a day that each possible pair of individuals was found associated “close together” at the same location, considering that this association occurred when the distance between them was less than 20 cm (i.e., approximately one body length of an adult amphisbaenian).

### Data analyses

We used a Linear Mixed Model (LMM) to analyze differences in the arcsine square-root transformed percentages of ‘movement activity’ (response variable) between the surveys made at different hours (‘time of day’; repeated-measures factor), between ‘classes’ of amphisbaenians (CORT-supplemented males, control males, and females; fixed factor). The ‘enclosure’ was included as a random factor to account for potential non-independence among individuals sharing the same enclosure and to control for any unmeasured environmental heterogeneity among enclosures. We included the log_10_-transformed SVL of each individual (‘body size’) as a continuous covariate. We also included in the models the interaction between ‘classes’ and ‘enclosures’ to test whether treatment effects were consistent across enclosures, as each enclosure contained individuals from all classes. Because each individual contributed repeated observations across days, within-individual dependence was accounted for through the repeated measures structure of the models. Including individual identity as an additional random effect did not change the results and was redundant with the repeated structure; therefore, it was not included in this or subsequent models. Pairwise post-hoc comparisons were made using Tukey’s tests.

For the analyses of movement and space-use variables (log_10_-transformed values of ‘total distance moved’, ‘number of movements’, ‘distance moved per movement’, ‘daily area covered’, and ‘total area covered’), we first calculated average values per individual across the 14 sampling days. These individual-level means were then used as response variables. Thus, the individual constituted the independent sampling unit in these analyses, and repeated daily observations were not treated as separate data points, thereby avoiding within-individual pseudoreplication. We examined in separate LMMs variation in these response variables between ‘classes’ of amphisbaenians (CORT-supplemented males, control males, and females; fixed factor), and between ‘enclosures’ (random factor), including the interaction between ‘classes’ and ‘enclosures’, and considering individual ‘body size’ as a continuous covariate.

To analyze the spatial relationships between individuals, the response variables were calculated as daily average values per dyad (i.e., each pair of individuals within an enclosure), rather than using raw survey-level observations. Thus, the statistical unit was the dyad-day average. We retained the dyad-level structure of the analyses to explicitly test our a priori hypotheses regarding differences between male-male and male-female relationships, and the specific effects of male CORT treatment on these distinct social interaction types, which would not be detectable using aggregated individual-level metrics. We used separate LMMs to compare the daily average ‘separation’ (log_10_-transformed) and ‘proximity’ (arcsin-transformed) of different combinations of two individuals (see below) as the response variables. In a first set of LMMs, we included as fixed factor two classes of pairs of individuals (‘sex’ effect: any male and any other male, M/M vs. any male and any female, M/F). Then, we compared in separate LMMs the effect of the experimental treatment of the males (control: M-; CORT-supplemented: M+) on the relationships between the different combinations of males (‘treatment’: M-/M- vs. M-/M+ vs. M+/M+), or between males and females (‘treatment’: M-/F vs. M+/F). We included in all the models the average log_10_-transformed SVL of the two individuals in the pair (‘body size’) as a continuous covariate. Because individuals participated in multiple dyads within the same enclosure, we included ‘enclosure’ as a random factor in all models to account for the shared social and environmental context and the resulting non-independence among dyads formed by the same set of individuals. We also included the interactions of ‘enclosure’ with ‘sex’ or with ‘treatment’ to test whether treatment or sex effects were consistent across enclosures.

## Results

### Movements

**Fig. 1:**
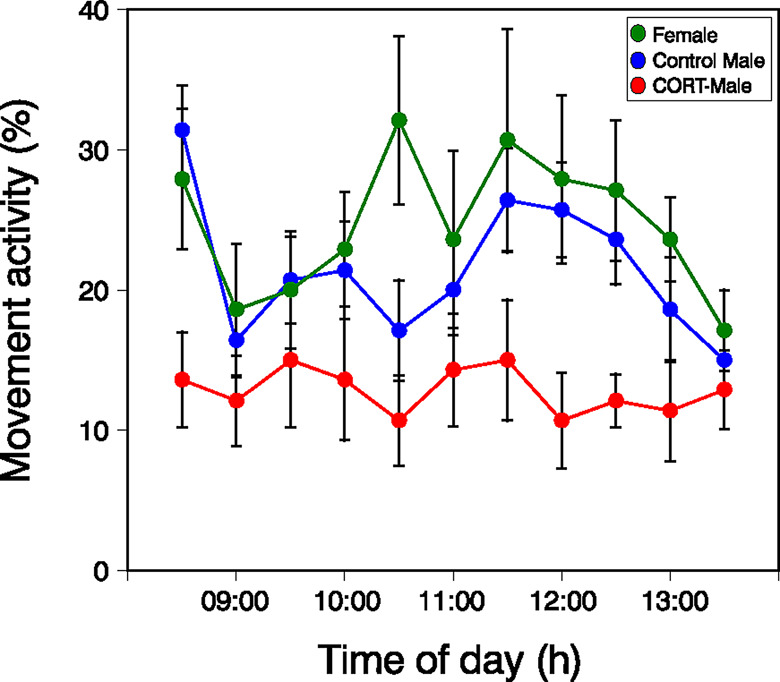
Effects of the CORT treatment on ‘movement activity’ (mean ± SE % of surveys made during the entire experiment in each of the 30 min time periods in which an individual had moved with respect to the previous survey) of female, control male, and CORT-supplemented male *B. cinereus* amphisbaenians in outdoor enclosures

Overall ‘movement activity’ of amphisbaenians during surveys was relatively low. On average, individuals changed position in only 19.7% of surveys (30 min intervals). However, ‘movement activity’ differed significantly among the three amphisbaenian ‘classes’ (LMM, F_2,14_ = 15.49, *p* = 0.0003). Neither ‘body size’ (F_1,14_ = 0.61, *p* = 0.45) nor ‘time of day’ (F_10,140_ = 0.66, *p* = 0.76) had significant effects (Fig. 1). Post-hoc tests showed that CORT-supplemented males moved less frequently than control males (Tukey’s tests, *p* = 0.0011) and than females (*p* = 0.0004). Control males and females did not differ (*p* = 0.75) (Fig. 1). ‘movement activity’ also varied among ‘enclosures’ (F_4,14_ = 6.17, *p* = 0.0003), but the ‘classes’ × ‘enclosures’ interaction was not significant (F_8,14_ = 1.98, *p* = 0.13).

**Fig. 2:**
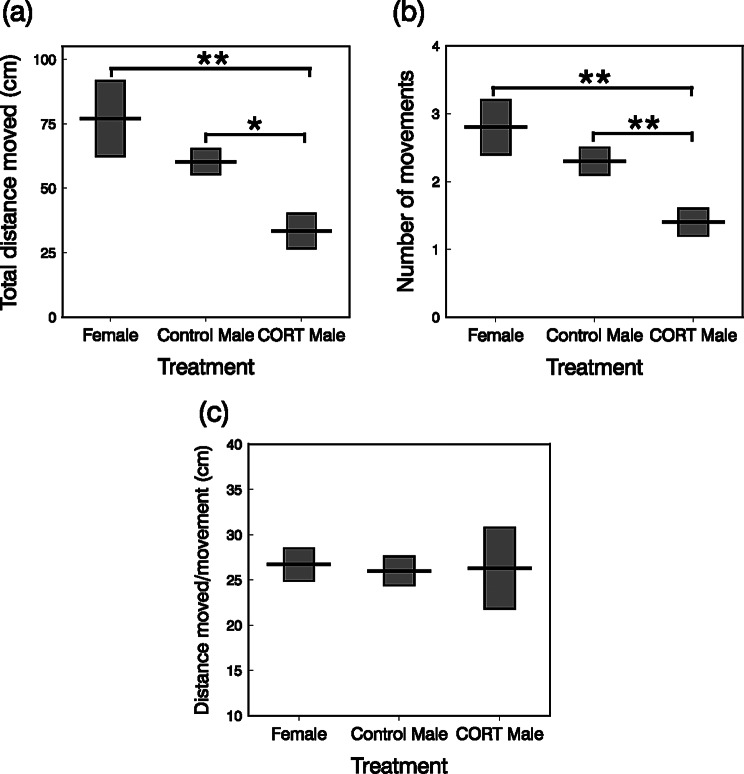
Effects of the CORT treatment on movements of female, control male, and CORT-supplemented male *B. cinereus* amphisbaenians in outdoor enclosures. Bars indicate mean ± SE of raw values of (**a**) ‘total distance moved’ (cm), (**b**) ‘number of movements’ (**n**), and (**c**) ‘distance moved per movement’ (cm). Asterisks above the bars indicate significant differences between means in post-hoc tests (*: *p* < 0.05; **: *p* < 0.01)

The ‘total distance moved’ also differed significantly among ‘classes’, with no effect of ‘body size’ or ‘enclosures’ (Table 1; Fig. 2a). CORT-supplemented males moved shorter distances than control males (Post-hoc Tukey’s tests, *p* = 0.018) and females (*p* = 0.007). Control males and females did not differ (*p* = 0.88) (Fig. 2a).

**Table 1:** Results of linear mixed models (LMMs) for the effect of ‘sex’ and CORT ‘treatment’ (classes of individuals; fixed factor), ‘body size’ (covariate), ‘enclosure’ (random factor), and the interaction between ‘enclosure’ and ‘classes’ on each of the different response variables that characterize movements and space use by *B. cinereus* amphisbaenians. Significant probabilities are marked in bold

	**df**	**Total distance moved**		**Number of movements**		**Mean distance moved**		**Daily area covered**		**Total area covered**	
		**F**	**p**	**F**	**p**	**F**	**p**	**F**	**p**	**F**	**p**
Classes	2	7.50	**0.0048**	7.41	**0.01**	0.25	0.78	11.46	**0.0004**	0.19	0.83
Body size	1	0.01	0.99	0.07	0.80	0.03	0.86	1.67	0.22	0.19	0.70
Enclosure	4	2.93	0.063	2.63	0.10	0.60	0.67	5.21	**0.006**	8.96	**0.0004**
Enclosure x Classes	8	0.70	0.71	3.44	**0.021**	0.08	0.99	0.33	0.94	0.36	0.93
Error	14										

The ‘number of movements’ showed the same pattern: significant differences among ‘classes’, no effects of ‘body size’ or ‘enclosures’, and a significant ‘classes’ × ‘enclosures’ interaction (Table 1; Fig. 2b). CORT-supplemented males moved on fewer occasions than control males (Tukey’s tests, *p* = 0.0004) and females (*p* = 0.0002). Control males and females did not differ (*p* = 0.74) (Fig. 2b). The significant interaction ‘classes’ × ‘enclosures’ suggested that that the magnitude of class differences varied across enclosures.

In contrast, the mean ‘distance moved per movement’ did not differ among ‘classes’ and was not related to ‘body size’ or ‘enclosure’ (Table 1; Fig. 2c).

### Space use

**Fig. 3:**
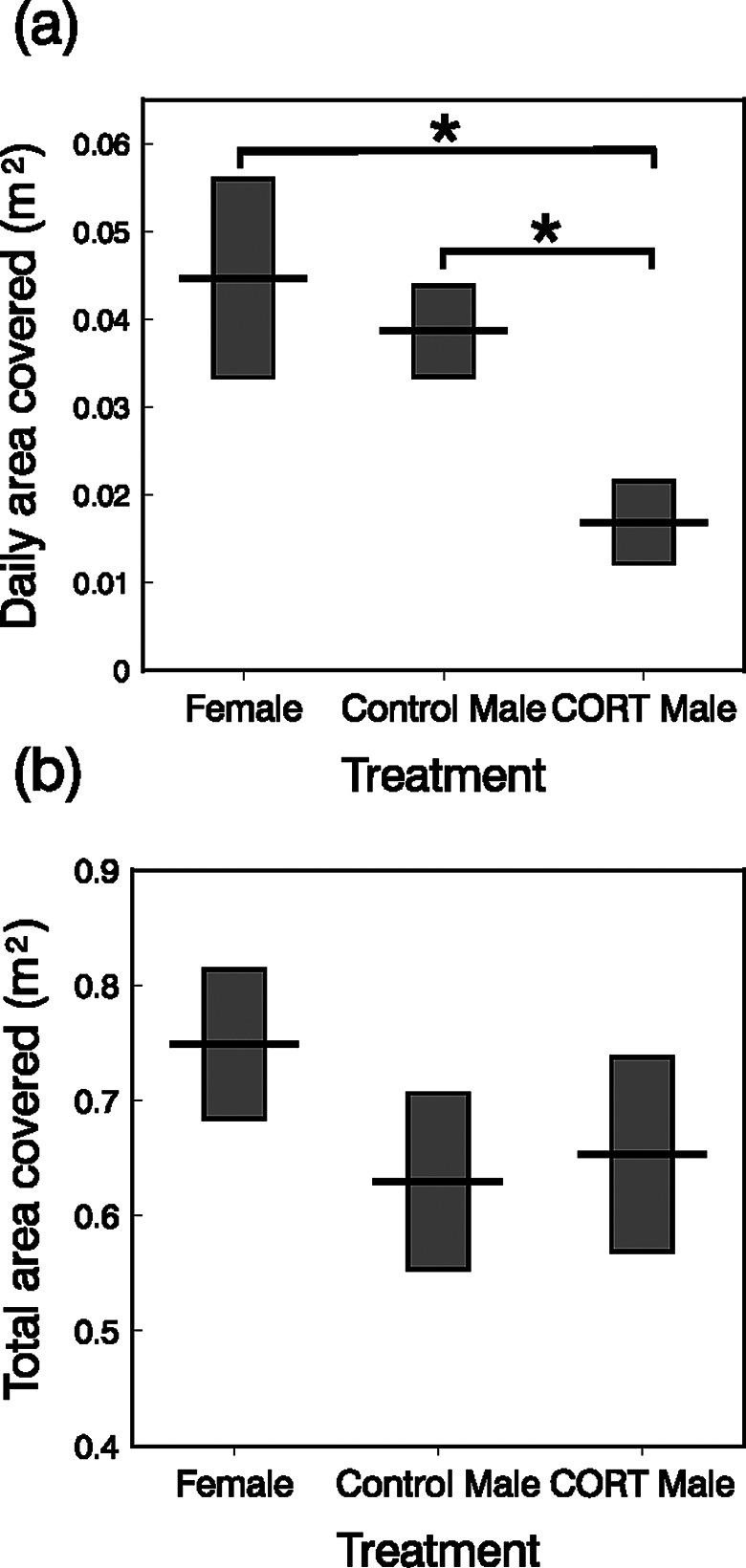
Effects of the CORT treatment on space use of female, control male, and CORT-supplemented male *B. cinereus* amphisbaenians in outdoor enclosures. Bars indicate mean ± SE of raw values of (**a**) ‘daily area covered’ (m^2^) during movements in a day and (**b**) ‘total area covered’ (m^2^) during the entire experiment. Asterisks above the bars indicate significant differences between means in post-hoc tests (*: *p* < 0.05)

The average ‘daily area covered’ differed significantly among ‘classes’, with no effect of ‘body size’ (Table 1; Fig. 3a). ‘Enclosures’ differed from one another, but the ‘classes’ × ‘Enclosures’ interaction was not significant. CORT-supplemented males covered significantly smaller daily areas than control males (Tukey’s tests, *p* = 0.028) and females (*p* = 0.04). Control males and females did not differ (*p* = 0.98) (Fig. 3a). Although enclosure effects were significant, post-hoc comparisons among enclosures were not (Tukey’s tests, 0.17 < *p* < 0.99 in all cases).

However, the ‘total area covered’ during the entire experiment did not differ significantly among ‘classes’ and was unrelated to ‘body size’ (Table 1; Fig. 3b). ‘Enclosures’ differed significantly, but the ‘classes’ × ‘Enclosures’ interaction was not significant. Post-hoc tests showed that only one enclosure differed significantly from two others (Tukey’s tests, *p* = 0.04 in both cases), while all remaining comparisons were non- significant (0.14 < *p* < 0.99).

### Spatial relationships between individuals

**Fig. 4:**
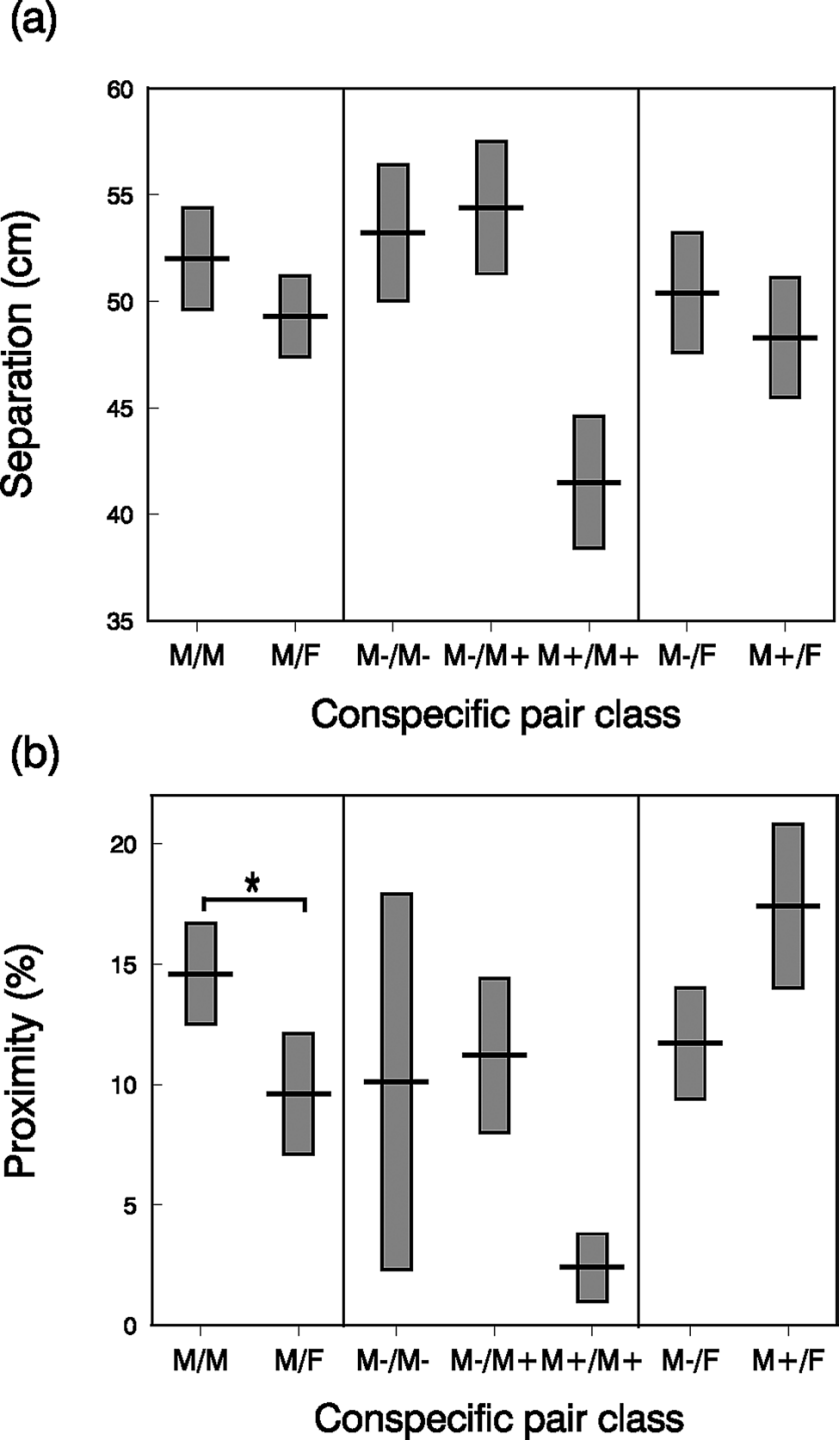
Effects of the CORT treatment on spatial relationships between female, control male, and CORT-supplemented male *B. cinereus* amphisbaenians in outdoor enclosures. Bars indicate mean ± SE of raw values of (**a**) ‘separation’ distance (cm) and (**b**) ‘proximity’ index (% of times located together) between classes of pairs of conspecifics (male: M; female: F; control male: M-; CORT-supplemented male: M+). Asterisks above the bars indicate significant differences between means (*: *p* < 0.05)

Mean ‘separation’ distances between a pair of males (M/M) or between a male and a female (M/F) did not significantly differ among classes, were not influenced by the male CORT treatment, and were not related to the average ‘body size’ of the pair (Table 2; Fig. 4a). Nevertheless, in the model for male-male relationships CORT-treated males tended to remain farther from any other males than control males, although this trend did not reach statistical significance.

**Table 2:** Results of linear mixed models (LMMs) for the effect of ‘sex’ (male: M; female: F) or CORT ‘treatment’ of the male (control: M-; CORT-supplemented: M+) (fixed factors), ‘body size’ (covariate), ‘enclosure’ (random factor), and the interaction between ‘enclosure’ and ‘sex’ or ‘treatment’ on each of the two response variables that characterize spatial relationships between pairs of *B. cinereus* amphisbaenians. Significant probabilities are marked in bold

		Separation		Proximity	
	df	F	p	F	p
Overall sex differences:					
Sex (M/M vs. M/F)	1	0.44	0.51	4.69	**0.036**
Body size	1	0.15	0.70	0.01	0.99
Enclosure	4	60.02	**0.0001**	10.66	**0.001**
Enclosure × Sex	4	0.03	0.99	0.37	0.83
Error	59				
Relationships between males:					
Treatment (M-/M- vs. M-/M+ vs. M+/M+)	2	3.21	0.09	1.17	0.36
Body size	1	1.24	0.28	1.32	0.27
Enclosure	4	2.56	0.08	1.63	0.22
Enclosure × Treatment	8	1.19	0.37	0.96	0.51
Error	14				
Relationships between males and females:					
Treatment (M-/F vs. M+/F)	1	0.31	0.61	2.37	0.20
Body size	1	0.01	0.95	0.26	0.61
Enclosure	4	5.38	**0.04**	3.93	0.06
Enclosure × Treatment	4	1.59	0.20	1.11	0.37
Error	29

Individuals were found within 20 cm of each other in only 12.4% of surveys (range = 0–56%). Males were significantly more often in close proximity to females (M/F) than to other males (M/M). However, this ‘proximity’ index did not depend on the male treatment or on the average ‘body size’ of the pair (Table 2; Fig. 4b).

Although some models showed significant differences among ‘enclosures’, the interactions with ‘sex’ or ‘treatment’ were not significant, indicating that ‘enclosures’ effects were consistent across pair types.

## Discussion

We found that the elevated CORT treatment decreased movement activity, distances moved, number of movements, and areas covered by CORT-supplemented male *B. cinereus*. However, we also found that although all males were more often in close proximity to females than to other males, there was no evidence of an effect of CORT supplementation of males on their spatial relationships (distance and proximity) with male or female conspecifics. Nevertheless, because studying fossorial reptiles under seminatural conditions imposes logistical constraints that limit our sample size, statistical power to detect subtle treatment effects may be reduced, and non-significant results should therefore be interpreted as absence of detectable effects rather than absence of biological differences.

CORT-supplemented male *B. cinereus* showed significantly decreased movement activity rates, and spent more time motionless at the same locations, which resulted in shorter distances moved and smaller areas covered during the day than those of control males. Nevertheless, when these CORT males moved, they travelled similar average distances in each burst of movement as controls. This reduction in movement activity observed several days after supplementation is likely a consequence of physiological carry-over effects associated with the cumulative activation of GC pathways during treatment, rather than evidence of a prolonged chronic physiological stress response [13, 14]. For example, increased CORT reduces the immune response in *B. cinereus* [42], and could also modulate metabolic rate and energy allocation [19]. CORT-treated males, with an altered physiological condition, might also have a low ability to move and cope with dangerous situations, such as a predator or an aggressive conspecific. Reducing activity and remaining longer in “safe” known places might help amphisbaenians to save energy and avoid potential encounters with predators or competitors in unexplored novel areas. These behavioral adjustments may also reflect CORT-mediated shifts in energetic strategy, consistent with the hormone’s role in regulating activity and resource use under challenging conditions.

Some studies exploring the impact of increasing CORT levels on locomotor activity in epigeal lizard species showed similar results. For example, elevated CORT in male side-blotched lizards (*Uta stansburiana*) reduced their activity, home range size, and aggressive behavior, likely because the effects of elevated CORT would put male lizards at a competitive disadvantage [22–24]. Additionally, since burrowing has high energetic costs for fossorial reptiles [54], CORT-supplemented male amphisbaenians may reduce underground movements to save energy that could be allocated to cope with the physiological effects of the stressor.

In contrast, CORT exposure had no effect on movement activity in some species of salamanders or birds [16, 25, 26]. Moreover, CORT-treated juvenile wall lizards (*Podarcis muralis*) increased the frequency of moving behavior [21], and although CORT-treated adult common lizards (*Zootoca vivipara*) did not increase movement, they became active earlier in the morning and spent less time immobile after emergence, suggesting that they increased their daily activity period [12]. This increase in movement activity could be interpreted as an adaptive behavioral response to avoid (e.g., by dispersing away from the origin of the stressor) or compensate for (e.g., by increasing feeding activity) the negative effects of natural perturbations [20, 55]. However, in some contexts, enhanced activity or dispersal could be negative if predation risk, or social and thermoregulatory costs increase [20, 26, 56]. This trade-off between avoiding the stressor and costs of dispersal could explain the different effects observed in different animals and situations. Differences in movement may also reflect underlying variation in metabolic rate, as CORT levels are known to covary with baseline metabolism independently of acute stress [19]. For example, for fossorial animals, burrowing movements are energetically costly [54]. Hence, adopting other strategies, such as reducing mobility instead of trying to evade stressful situations by increasing movements can be advantageous for underground-dwelling species.

In our experiment, the reduced movement activity of CORT-supplemented *B. cinereus* males might also be partly aimed at reducing the probability of unexpected encounters with other males, which may react by biting the intruder individual aggressively [57]. However, our results did not match the initial prediction that, to avoid these costly agonistic interactions, CORT-supplemented males should have space-use strategies to avoid being closer to other males. This objective was not achieved even after CORT males reduced the size of their daily areas used and, potentially, exhibited a tendency to remain farther from other males compared to control individuals. Although it might initially seem that underground environments make it difficult for individuals to detect the precise location of nearby conspecifics, and thus to avoid aggressive encounters [57], studies on chemical sensing show that amphisbaenians can use information in chemical cues from substrate scent marks of conspecifics to identify and avoid areas highly used by competitively superior individuals [58]. While agonistic encounters may be more costly for CORT-supplemented males, this ability to detect chemical cues likely allows males to reduce the risk of conflict, which could explain why all males were generally found closer to females than to other males. Accordingly, the elevation of CORT does not appear to affect the spatial patterns of males relative to other males.

The reduced movements and small daily areas covered by CORT-supplemented male *B. cinereus* might have negative reproductive consequences if this change in behavior decreased the probability of encountering females. However, although CORT-treated males covered smaller areas on a daily basis, their total area covered across the entire experiment did not differ from controls. This pattern indicates that CORT affected the short-term rate of space use rather than the final extent of space explored. Because the experiment spanned multiple days, even individuals with reduced daily activity eventually traversed most of the enclosure, resulting in similar total spatial coverage and potentially similar rates of encounters with females across treatments. Moreover, our results did not confirm the prediction that females might prefer to be closer to non-CORT-supplemented control males, which might be better potential mates compared with CORT-treated males. We found no evidence for CORT treatment effects (under the current design and sample size) on the mean distance between a male and a female, or on the time they spent in close proximity. Similarly, CORT supplementation in male side-blotched lizards (*U. stansburiana*) reduced aggressive behavior but did not affect male courtship or copulatory behavior [22]. Because increased CORT levels may affect the physiological condition of supplemented *B. cinereus* males (e.g., by decreasing their immune response) [42], we had expected that this poorer state would be considered by females when deciding to stay close to a given individual male that could be a potential mate. However, a previous experiment showed that female *B. cinereus* did not prefer scent marks of a male as a function of the CORT manipulation of that male, but rather of its actual physiological condition, reflected by its individual immune response [42]. This interindividual variation may arise because elevated CORT can affect individuals differently [15], depending on their underlying physiological condition and their capacity to cope with the energetic and immune challenges associated with stressors [47]. Therefore, we did not find evidence that the behavioral changes exhibited by CORT-treated males in our experiment could have imposed substantial immediate reproductive costs.

Although seminatural enclosures in our experiment were designed to be homogeneous, they inevitably reduce the environmental heterogeneity present in natural habitats. As a result, movement patterns observed here may underestimate the variability that individuals experience in the wild, where habitat structure, resource distribution, and social context are more complex. However, the results may still provide an approximation ofgeneral activity levels and movement rates that could also be expected under natural conditions. For example, field observations of movement patterns in another similarly sized amphisbaenian species (*T. wiegmanni*) [35] revealed similarly low levels of natural daily activity (35% of days without detectable movement),with a mean daily displacement (80 cm/day) comparable to that of control *B. cinereus* in our experiment. In contrast, under natural conditions, *T. wiegmanni* individuals covered much larger daily areas (mean = 5000 cm^2^) [35] than *B. cinereus* in our experiment (mean = 339 cm^2^). Although one control male showed an exceptional daily maximum value of 7052 cm^2^, excluding this observation from the analyses did not change the overall low mean values or the direction and significance of the effects. However, in general, these daily areas covered by *B. cinereus* amphisbaenians were also much smaller than the potential total area available that could have been used in an enclosure (approx. 18,000 cm^2^). Therefore, the small areas covered and the observed changes between treatments should not be simply attributed to space restrictions due to the size of the enclosure, even if all individuals in an enclosure were forced to use the space such that there was no overlap at all between their areas. The differences in the environments occupied by these two species might instead explain their different home ranges.

The results of our experiment support our initial hypothesis that sustained elevations in GCs reduce movement rates and spatial exploration in fossorial reptiles. Increased CORT levels, whether arising from environmental disturbances, energetic constraints, or repeated stressors, may promote more conservative movement strategies, consistent with the reduced activity and smaller daily areas observed in CORT-supplemented males. Although our treatment did not aim replicating a specific environmental disturbance, these findings may have important implications for the conservation of soil biodiversity. The soil environment is increasingly affected by anthropogenic perturbations associated with global change, such as increased temperatures, drought, erosion, and pollution [29]. These perturbations may increase the stress response of soil animals [30–32, 37], particularly in species with lower dispersal ability [35], which limits their capacity to “escape” from the origin of the perturbation.

In fossorial reptiles, the negative consequences of elevated CORT may stem more from energetic constraints of their biology rather than only from direct harmful effects of GCs. Underground movement is energetically costly, and opportunities for energy acquisition are limited, so GC-mediated reductions in activity may restrict foraging, mate searching, or territory exploration. Consequently, elevated CORT levels in response to soil perturbations could disproportionately affect fossorial species by intensifying these behavioral trade-offs. Fossorial animals can show behavioral adaptations, such as changes in movement activity, that partially mitigate the effects of elevated GC levels, buffering potential fitness costs. However, if stressful conditions persist or intensify, the associated costs of these behavioral adjustments may become too high, leading to significant fitness consequences. Our findings therefore highlight how repeated GC activation may shape behavioral adjustments with ecological costs, even in the absence of chronic stress pathology. To better understand how global change may affect soil biodiversity, future studies should examine, through observational and experimental approaches, how different types and intensities of soil perturbations influence the behavior, physiological condition and fitness of fossorial animals.

## Data Availability

The datasets supporting the conclusions of this article are available in the Figshare repository, 10.6084/m9.figshare.30399025.
